# Antioxidant and Anticancer Activities of *Barleria longiflora* L. f. From Siriya Kalvarayan Hills

**DOI:** 10.1155/sci5/8055358

**Published:** 2025-05-28

**Authors:** Panjatcharam Varadharasu, Kandavel Dhandayuthapani, Pushparaj Annadurai, Vishal Ahuja, Shikha Chauhan, Kalpana Tilak, Balamurugan Sudarrajan, GholamReza Abdi, Deepak Sharma

**Affiliations:** ^1^Department of Botany, Thanthai Periyar Government Arts and Science College (Autonomous), Affiliated to Bharathidasan University, Tiruchirappalli 620023, Tamil Nadu, India; ^2^Department of Botany, Affiliated to University of Madras, Government Arts College, Nandanam, Chennai 600035, Tamil Nadu, India; ^3^Department of Medicinal Botany and Pharmacognosy, Nanda Siddha Medical College and Hospital (Affiliated to Dr. M. G. R. Medical University, Tamil Nadu), Erode 638052, India; ^4^Department of Biotechnology, University Institute of Biotechnology, Chandigarh University, Mohali 140413, Punjab, India; ^5^Department of Biotechnology, University Centre for Research & Development, Chandigarh University, Mohali 140413, Punjab, India; ^6^Department of BioTechnology, Persian Gulf Research Institute, Persian Gulf University, Bushehr, Iran; ^7^Department of General Surgery, Saveetha Medical College and Hospital, Saveetha Institute of Medical and Technical Sciences, Thandalam, Chennai 602105, Tamil Nadu, India

**Keywords:** anticancer, antioxidant, *Barleria longiflora*, in silico analysis, phytochemicals

## Abstract

*Barleria*, the third largest genus of the Acanthaceae family, carries a distinct position due to cultural as well as economic significance. Its aerial parts including stem, flowers, leaves, and underground part roots have been used in ancient civilizations as ornament, food, and for religious activities since ages. The presence of diverse phytochemicals is accountable for diverse healthcare applications including analgesic, antioxidant, and antimicrobial. In current work, previous knowledge is exploited as the foundation for the evaluation of *Barleria longiflora* leaves from the Siriya Kalvarayan hills region against lung cancer. The crude ethanolic extract of leaves has shown significant anti-inflammatory and antioxidant activity. Leaf extract has considerable in vitro free radical scavenging activity in DPPH and ABTS assay, *i.e.*, 62% and 64%, respectively, in comparison to L-ascorbic acid (100 g.mL^−1^). Leaf extract has also shown commendable cytotoxicity against A549 cells (IC_50_: 71.00 μL.mL^−1^). Gas chromatography-mass spectrometry identified around 38 phytochemicals including stigmasterol, resorcinol, and 3,4-anhydro-d-galactosan*. In silico* analysis identified good binding molecules through molecular docking studies, especially stigmasterol having significant Ki values against the cancer receptors such as PI3K, mTOR, and ERβ. The phytochemicals from *Barleria longiflora* have shown commendable antimicrobial and anticancer activities which is also supported with in silico analysis. The compounds responsible for anticancer activity can become major ingredient for drug formulation after trials.

## 1. Introduction

Plants have been used as medicine for centuries due to the lower frequency of side effects. Different traditional healthcare systems like Ayurveda in India [1], traditional Chinese medicines [2] and the East-Mediterranean region [3], Siddha, Unani, and Homeopathy [4] etc., have used plant-based formulations for health care, disease prophylaxis and treatment. Ancient literature like Vedas described numerous healthcare formulation including concoctions, fumes, infusions, tincture, decoctions, and teas [5, 6] prepared with plants and herbs due to the presence of abundant phytochemicals including amines, phenols [7], flavanols [8], and isoprene derivatives [9] that are accountable for their antioxidant, antibacterial, anticarcinogenic, anti-inflammatory, antidiabetic [10, 11], antitumor, antileprosy, and antiviral effects [12–15]. Currently, many commercial products and drugs are formulated using either natural phytochemicals or their derivatives.

In context to biodiversity, Indo-Himalayan region [16, 17] and Western Ghats' forest and hills are among the richest natural resources regarding flora and fauna [18]. These plants carry a significant role in ancient human civilization, culture, religious beliefs, and ceremonies [19]. *Barleria*, one of the largest genera, belongs to Acanthaceae family distributed in Southeast Asia known for widespread pharmacological applications in curing inflammations, pain, leukemia, cancer, tumor, glycemia, amoeba, viral, and other microbial infection [12]. *Barleria longiflora* is one of the shrub species, widely distributed in “Western Ghats.” It is a tiny shrub with 1-2 m height with oppositely arranged pointed hairy leaves. The flower's tube is white with a purplish color which is 8-9 cm long and narrow [20]. Various species of *Barleria* have shown conducive results for pharmacological properties [12]; however, *B. longiflora* has not been explored much for such analysis.

On the other hand, carcinogenesis incidents especially lung cancer cases have increased tremendously due to pollution and increased exposure to chemicals and radiation. World Cancer Research Fund International has identified lung cancer as the second most common cancer across the globe with more than 2.2 million cases alone in 2020. Hungary was the most affected country in 2020 with 10, 274 cases followed by Serbia, France, and New Caledonia [21]. Therefore, the current work was intended to examine the potential of *B. longiflora* as a potent source of bioactive phytochemicals with cytotoxic and inhibitory potential efficacy against lung cancer. The work combines the empirical and traditional knowledge with advanced statistical and in silico analysis for target specificity and ADMET profiling.

## 2. Materials and Methods

### 2.1. Collection of Plant Part (Leaves)

*B. longiflora* is prominently present in South Eastern Ghats of India. For the work, plant leaves were collected from Siriya Kalvarayan hills, part of South Eastern Ghats, Kallakurichi District (Tamil Nadu), India, in December-January. The collected sample specimen was identified at Department of Botany, Government Arts College, Nandanam, Chennai-600035. The specimen has been assigned the ID NO: GACNBOT101.

### 2.2. Extract Preparation

Phytochemical extraction from *B. longiflora* leaves was done with the Soxhlet apparatus using polar and nonpolar solvents, viz., aqueous, hexane, ethanol, acetone, methanol, and chloroform as per established protocol [22, 23].

### 2.3. Phytochemical Analysis

Quantitative analysis of phytoconstituents in the ethanolic leaf extract of *B. longiflora* was determined by different conventional methods. Chlorophyll and carotenoids were quantified by the Arnon method [24]. Total sugar and lipid content in extract were assessed by the DuBois method [25] and gravimetric method [26], respectively. Protein and amino acid concentration in extract was quantified by the “Lowry method” [27] and “ninhydrin assay” [28], respectively. Phenolics, flavonoids, and tannins were quantified by using the Folin–Ciocalteu method [29].

### 2.4. Antioxidant Activity of *B. longiflora* Leaf Extract

#### 2.4.1. DPPH Assay

Antioxidant activity of *B. longiflora* leaf extract against free radicals was estimated by DPPH assay which traces transfer of electrons that develops a violet color solution in methanol [30]. Crude leaf extract at varying concentrations (10–100 μg.mL^−1^) and an equal volume of fresh DPPH (0.1 mM methanolic solution) were mixed vigorously and kept in dark for 30 min at room temperature. The scavenging activity resulted in disappearance of violet color and was estimated at 520 nm. For comparison, negative control (distilled water) and positive control (L-ascorbic acid) were used. The scavenging activity of *B. longiflora* leaf extract was measured using the following equation:

(1) $$\begin{array}{c} \text{Inhibition }\left(\%\right)=\left(\frac{A_{c}-A_{s}}{A_{c}}\right)\times 100, \end{array}$$

where *A*_*c*_ and *A*_*s*_ denote the absorbance of positive control and sample, respectively.

#### 2.4.2. 2,2′-Azino-Bis(3-Ethyl-Benzothiazoline-6-Sulfonic Acid) (ABTS) Assay

The antioxidant efficacy of *B. longiflora* was tested by ABTS radical cation decolorization assay [31, 32]. For analysis ABTS (7 mM aqueous solution) and potassium persulfate (2.45 mM aqueous solution) mixed in equal amount and kept at room temperature in dark for 12–16 h.It generatesABTS radical cations (ABTS^o+^). ABTS^o+^ solution was diluted with ddH_2_O to attain OD of 0.700 at 734 nm. ABTS^o+^ fresh solution (3.995 mL) was mixed with 5 μL *B. longiflora* leaf extract and kept again for 30 min followed by absorbance recording at 734 nm. The scavenging potential was determined using equation (1).

### 2.5. Cytotoxic Activity of *B. longiflora* Leaf Extract

Cytotoxicity is the ability to be toxic to cells. The cytotoxicity of *B. longiflora* extract was studied on the A549 cells following MTT assay [33]. A549 cells, procured from NCCS, Pune (India), were maintained in Dulbecco's Modified Eagle Medium (DMEM) with 10% FBS and 100 μg.mL^−1^ of both streptomycin and penicillin. The cells were incubated at 37°C with 5% CO_2_ in a CO_2_ incubator. For cytotoxicity assay, A549 cells were transferred to a 24-well plate (1 × 10^5^ cells per well) and were grown to confluence at 37°C with 5% CO_2_. Different concentrations of extract were added to each well and incubated again for 24 h. After incubation, excess plant extract was removed by from well and washed with PBS (pH 7.4). For cell viability analysis, MTT (100 μL; 0.5%) was added to each well, incubated for 4 h and mixed with 1 mL DMSO. The absorbance was recorded at 570 nm and compared with DMSO and commercial drug as negative and positive control. Cell viability was calculated by the following equation:

(2) $$\begin{array}{c} \text{Cell viability}\left(\%\right)=\frac{\text{Absorbance of treated cells}}{\text{Absorbance of control cells}}. \end{array}$$

### 2.6. Anti-Inflammatory Activity

In vitro anti-inflammatory potential of *B. longiflora* extract was determined by the Human Red Blood Cell (HRBC) method [34]. Blood of healthy volunteers was mixed in equal volume with fresh Alsevers solution (citric acid (0.9%, w/v), sodium chloride (0.72%, w/v), dextrose (2.0%, w/v) and sodium citrate (0.8%, w/v)). Packed red blood cells from mixture was collected by centrifugation at 4°C for 15 min at 10,000 rpm. After centrifugation, sample washed and suspended in isosaline solution. The HRBC suspension was used for estimation of the anti-inflammatory activity of the leaf extract. Varying concentrations of leaf extract were separately mixed with phosphate buffer (2.0 mL), HRBC suspension (0.9 mL), and hyposaline (4.0 mL) and incubated for 30 min at 37°C. Solution without leaf extract served as control. The mixture was centrifuged at 3000 rpm and pellet was considered to estimate hemoglobin content by spectrophotometer at 620 nm [35]. Hemolysis (%) was calculated by the following equation:

(3) $$\begin{array}{c} \text{Hemolysis}\left(\%\right)=\frac{{\text{OD}}_{\text{sample}}}{{\text{OD}}_{\text{control}}}\times 100, \end{array}$$

where OD_sample_ and OD_control_ denote absorbance of test sample with plant extract and control.

### 2.7. Gas Chromatography-Mass Spectrometry (GC-MS) Analysis

The phytochemical constituents in leaf extract were identified using GC-MS (Perkin Elmer Clarus 500). Phytochemical profiling of leaf extracts of was carried out with GC-MS system equipped with Phenomenex Luna PFP analytical capillary column (30 m × 0.25 mm × 0.25 μm) and flame ionization detector. The analysis was conducted with carrier gas (He) flow rate of 1 mL.min^−1^, 10 μL injection volume injected at 280°C (injection port temperature), and stationary phase temperature ranging from 60°C to 300°C (10°C.min^−1^). Mass spectrum of extract was obtained with full scan mode (40–450 Daltons) and identification was done with the National Institute of Standards and Technology (NIST) library [36].

### 2.8. Docking Analysis

The structures of phytochemicals, identified by GC-MS, as well as receptor proteins for lung cancer, e.g., 5NGB (PI3K), 4JT6 (mammalian target of rapamycin (mTOR)), ERβ, and 3PP0 (human epidermal growth factor receptor-2 (HER-2)), were retrieved from the Protein Data Bank (PDB) and PubChem repositories for docking analysis using AutoDock Tools (ADT) [37, 38]. Different commercial inhibitors are used as control drugs for docking studies, e.g., taselisib and dactolisib for 3NG, rapamycin and ridaforolimus for 4JT6, toremifene for 3OS8, and neratinib and lapatinib for 3PP0.

## 3. Results and Discussion

The leaf extracts of *B. longiflora* contained sugar, catechin, flavonoids, triterpenoids, saponin, tannins, anthraquinone, amino acids, sterol, and carbohydrates, according to the results of phytochemical screening of the extracts (Table 1). The *B. longiflora* extracts in methanol, ethanol, and chloroform were particularly effective suppliers of various types of chemicals. This shows that these solvents' strong polarity makes them useful for isolating biologically active molecules, and flavonoids were found in the leaf extracts of the plant in chloroform and acetone.

Among all the solvents, ethanolic extract has shown higher potential bioactivity and was hence selected for further characterization. The leaf extracts of *B. longiflora* contained sugar, catechin, flavonoids, tannins, triterpenoids, anthraquinone, saponin, amino acids, and sterol (Table 2), which may be responsible for bioactivity.

### 3.1. Antioxidant Activity Analysis of Plant Extract

**Figure 1:**
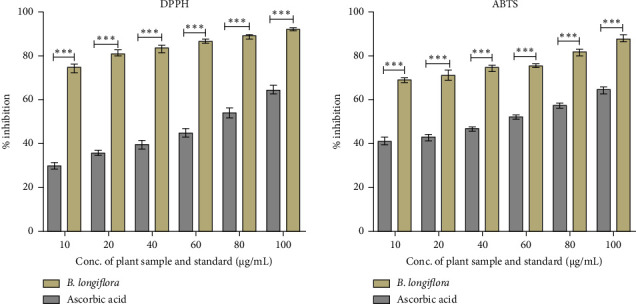
Antioxidant activity of *B. longiflora* ethanolic leaf extract.

The majority of natural antioxidants originated from plants such as herbs, fruits, vegetable, and spices which contain mostly phenolic compounds, alkaloids, steroids, vitamins, and carotenoids. In the present investigation, ethanolic plant leaf extract of *B. longiflora* has shown considerable antioxidant properties, *i.e.*, 30%–65% (DPPH assay) and 41%–63% (ABTS radical cation) at 10–100 μg.mL^−1^ concentration range in comparison to ascorbic acid (75%–95% in DPPH and 70%–90% in ABTS assay) (Figure 1).

### 3.2. Cytotoxicity of *B. longiflora* Leaf Extract Against A549 Cells

**Figure 2:**
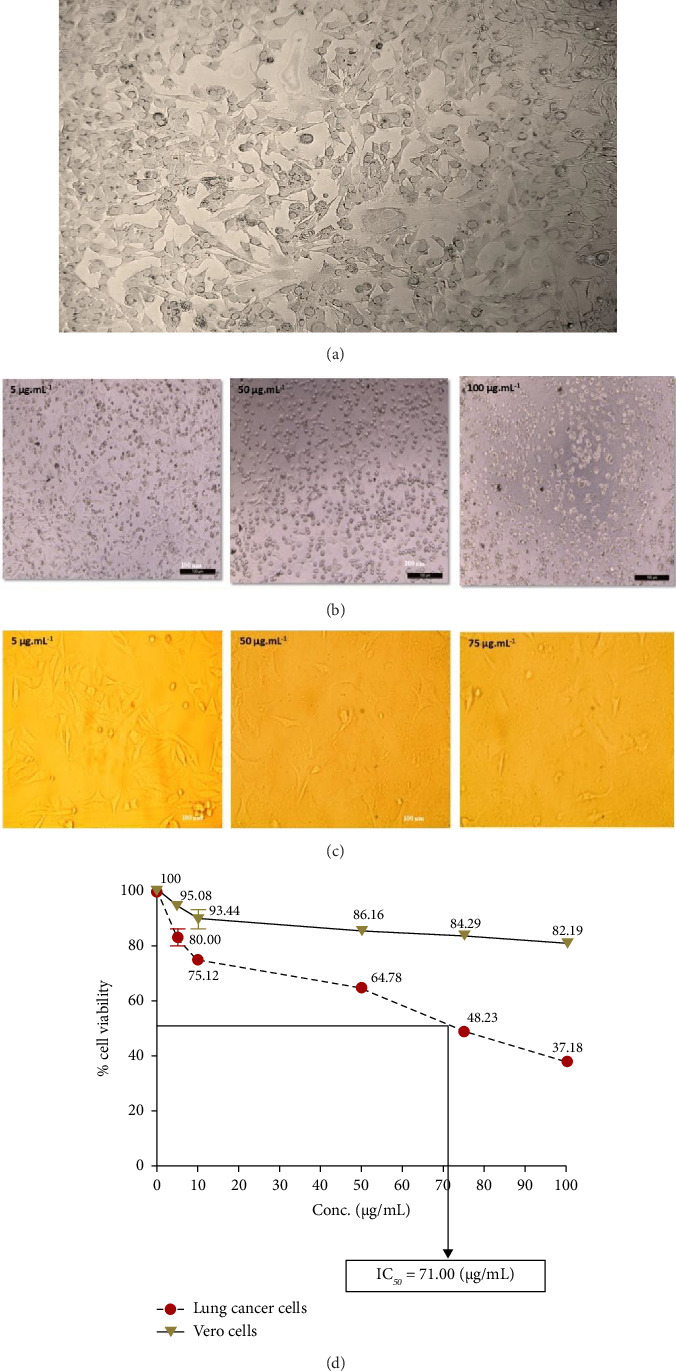
Cytotoxic effect of *B. longiflora* leaf extract against lung cancer cells. (a) A549 lung cancer cell line control. (b) A549 cell lines treated. (c) Vero cells treated. (d) Cytotoxicity curve and IC_50_ value.

The anticancer or cytotoxic effect of *B. longiflora* extract against A549 cell line was investigated with ethanolic extract. Over the past 3 decades, cytotoxicity studies have been crucial in the development of new anticancer formulations. The cytotoxic action of *B. longiflora* extract demonstrated that plant extract inhibited the proliferation of cancer cells in dose and time-dependent manner (Figures 2(a), 2(b), and 2(c)).

Compared to cancer cells, the cytotoxic study analyzed showed a considerable reduction in cell viability (A549). The percentage of cell viability of A549 revealed a clear declining trend in a dose-dependent manner. Similar patterns were seen with Vero cells at concentrations of 0–20 g.mL^−1^, with IC_50_ values for lung cancer cell lines less than 71.00 μL.mL^−1^ (Figure 2(d)). Consequently, an extract from *B. longiflora* may be utilized to treat cancer in people.

### 3.3. Anti-Inflammatory Analysis of *B. longiflora* Extract

**Figure 3:**
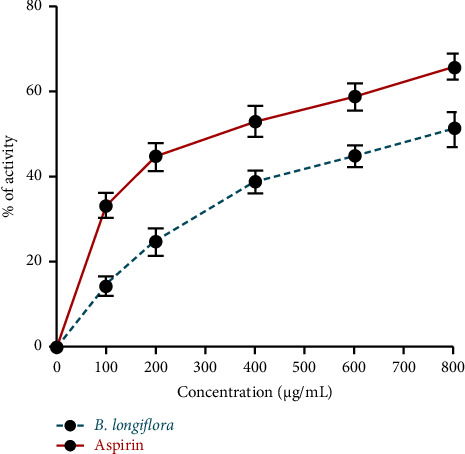
Anti-inflammatory potential of *B. longiflora* ethanolic extract of leaves.

*B. longiflora* leaf extract, used in the current investigation, has shown anti-inflammatory activity as a clear declining trend in inflammatory markers was observed in a dose-dependent manner. Similar patterns were seen with aspirin cells at concentrations of 0–800 μg/mL (Figure 3).

### 3.4. GC-MS Analysis

The chemical profiling of *B. longiflora* leaf extract GC-MS has identified a total of 38 compounds (Table 3). Among the listed compounds, some compounds are critical for bioactivity of plant leaf extract while some are part of structural components. Among the identified compounds, 3 compounds were dominant with peak area, e.g., linoleic acid (16.84%), palmitic acid (11.69%), phthalic acid (8.09%), solanesol (4.13%), and palmitoleic acid (2.52%). In the current work as well, out of 38, only 7 were major compounds. Based on the available literature, all the compounds were examined for their bioactivity and used for docking with the selected receptors, which play a key role in lung cancer.

### 3.5. In Silico Docking Studies

**Figure 4:**
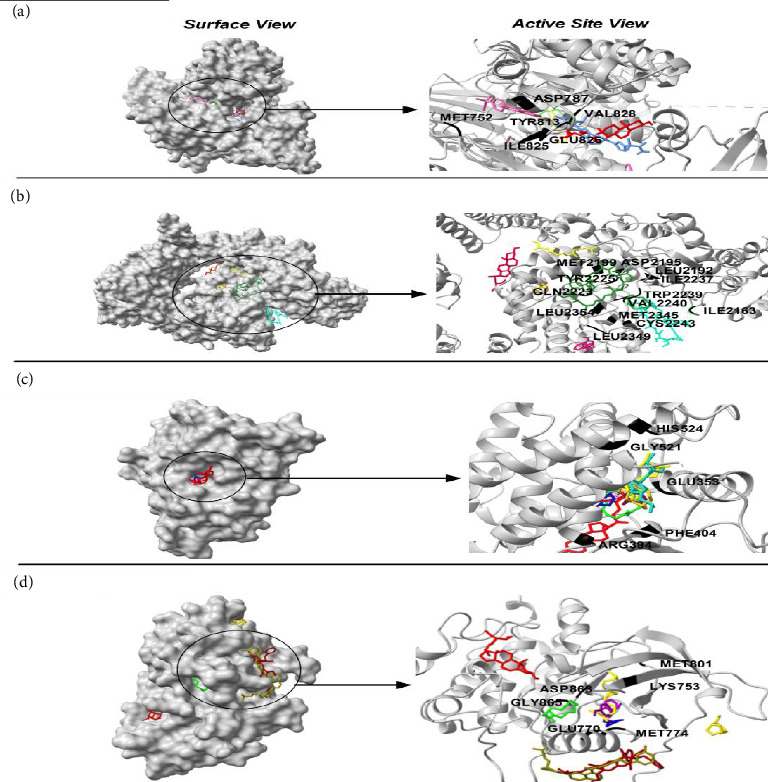
Interaction site for phytochemicals with target receptors. (a) 5NGB (PI3K). (b) 4JT6 (mTOR). (c) 3OS8 (ER beta). (d) 3PPO (HER-2).

The phytochemicals identified from *Barleria longiflora* leaf extract by GC-MS may be responsible for the bioactivity specifically anticancer activity but to get the type of interaction of phytochemicals with target receptors, docking analysis was conducted. Among 38 phytochemicals, only 8 have shown some affinity with the target receptors and hence were selected for further interaction studies (Figures 4(a), 4(b), 4(c), and 4(d) and Table 4).

Table 4 summarizes the interaction profile of selected phytochemicals and compares them with commercially available inhibitors available in the market, i.e., taselisib and dactolisib for 5NGB, rapamycin and ridaforolimus for 4JT6, toremifene for 3OS8, and neratinib and lapatinib for 3PP0. For 5NGB, dactolisib has the lowest binding energy along with an inhibitory constant of 0.004 μM. In comparison to commercial inhibitors, only one phytochemical “azacyclotridecan-2-one” has shown compatible results with a binding energy of −8.43 kcal/mol; however, the inhibitory constant was much higher (0.665 μM). Only one phytochemical “stigmasterol” has shown potential efficacy against the other three protein targets, i.e., 3PP0, 3OS8 chain A, and 4JT6 chain A. Against 4JT6 and 3OS8, stigmasterol has lower binding energy and lower inhibitory constant which suggest that the effective concentration of phytochemical is much lower in comparison to commercial inhibitors.

#### 3.5.1. PI3K

PI3K has a crucial role in several cancers and is a downstream effector of receptor tyrosine kinases such as insulin receptor and HER-2, which transduce growth factor signaling [62]. PI3K governs phosphatidylinositol-3,4,5-triphosphate (PIP3) production and activates Akt (protein kinase B) and other kinases. This protein has a “p85” regulatory domain and “p110” catalytic domain. “PI3K-Akt-mTOR” pathway reigns cellular longevity, cellular proliferation through nutrient uptake (as well as anabolism) and finally, increases cell survival through apoptosis inhibition. The “PI3K/Akt/mTOR pathway” is commonly dysregulated in almost all human cancers, and hence the proteins of this pathway are prime targets of anticancer therapeutic regimes [63, 64].

Often, inhibitors of this pathway decrease cellular proliferation and increase cell death. Since cancer cells achieve immortality, many therapeutic molecules are directed at the activation of apoptosis. In this work, some of the phytochemicals identified from *B. longiflora* extract with their favorable binding energies and low Ki values are compatible with control compounds such as TLB and DLB. As mentioned above, azacyclotridecan-2-one (ACT) and stigmasterol (SSL) have minimum binding energy among all phytochemicals (−8.43 and −8.06 kcal/mol, respectively) but higher than the commercial inhibitors like taselisib and dactolisib (−9.34 and −11.36 kcal/mol, respectively). In terms of inhibitory constant and effective dosage,the quantity of compounds is minimal in the case of dactolisib (0.004 μM) and taselisib (0.143 μM) which is much lower in comparison to 0.665 μM (azacyclotridecan-2-one) and 1.23 μM (stigmasterol).

**Figure 5:**
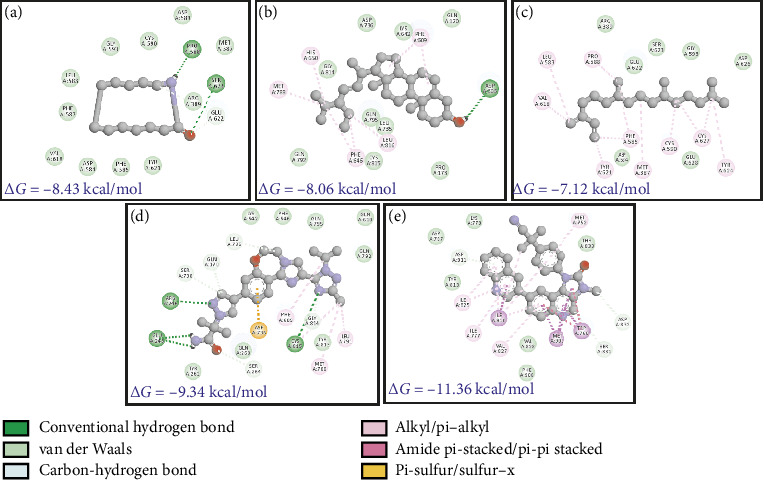
Interaction of PI3K receptor with phytochemicals. (a) ACT. (b) SSL. (c) NPD. (d) TLB. (e) DLB.

The kinase domain of human p110 is located between residues ∼696 and 1068. In PI3K p110α, the key residue involved in phosphoryl group transfer reaction is Lys802. Residues involved in substrate stabilization in PI3K which line the binding pocket are 941-KKKKFGYKRER-951 [65]. The drug XL765 was found to dock at the site of natural ligand LXX in the kinase domain of p110 and some common residues were found in interactions of both the inhibitor XL765 and the natural ligand [66]. In our analysis, we found that diverse kinds of interactions were proffered by PI3K in docking the plant compounds. The best interactions comprised of bonding and nonbonding interactions; in SSL association with PI3K, a hydrogen bond between ASP606 and PI3K p110 can be seen (Figures 5(a), 5(b), 5(c), 5(d), and 5(e)); also, other interactions such as pi-alkyl or alkyl (as well as nonbonded interactions such as van der Waals) are seen. In RCL binding, pi-stigma was observed. Strangely, the commercial compounds did not show high-affinity interactions with PI3K.

#### 3.5.2. mTOR

**Figure 6:**
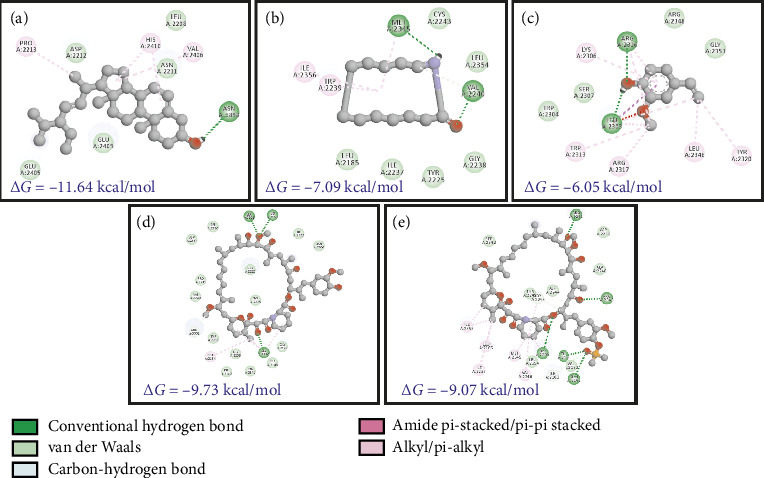
Interaction of mTOR receptor with phytochemicals. (a) SSL. (b) ACT. (c) MVP. (d) RMP. (e) RFL.

mTOR is a PI3K-related Ser/Thr kinase which is responsible for cell growth through direct or indirect phosphorylation of ∼800 proteins [67]. Since the protein had two distinct chains (A and C in PDB ID: 4JT6), docking was done separately for the two chains. Against the larger chain A, the plant compounds showed better Δ*G* values and were able to interact better with chain A, as evidenced by the favorable bonding (conventional H-bonding of SSL and Pi-alkyl bonding of RCL and several other forces such as van der Waals). Comparatively, the control compounds (RMN and RFL) had better dock scores (−9.07 to ∼−9.73) which are responsible for the existence of several carbon-hydrogen bonds with the protein (Figures 6(a), 6(b), 6(c), 6(d), and 6(e)). The predicted Ki values for the docked plant compounds were much lower than those of controls (Table 5).

Stigmasterol (SSL), Resorcinol (RCL), and 3,4-Anhydro-d-galactosan (ADG) have Ki values of 0.002 μM, 418.0μM, and 629.69 μM, respectively with mTOR. On the other hand, the smaller subunit (chain C) was found to interact with the control drugs RMN and RFL with binding scores (Δ*G*) of −9.73 and −9.07 kcal/mol, respectively (shown in red, Figures 6(d) and 6(e)), yielding predicted Ki values in both nanomolar and micromolar ranges. However, from literature survey, the kinase site of mTOR possesses two lobes, the KD N lobe and the KD C lobe, and the kinase domain of mTOR is located in chain A. Key residues of mTOR reported earlier for docking of substrates as well as inhibitors are located in the KD N lobe and the residue numbers are Asp2195, Asp2357, Tyr2225, Ile2163, Pro2169, Leu2185, Asn2343, Lys2187, Glu2190, Asp2357, and Asp2338 [68, 69]. Compared to the interactions between mTOR chain A and C, since the kinase activity of the protein is present in chain A, it is assumed that the docking results obtained for *B. longiflora* compounds with chain A could point to the ability of the compounds to inhibit the kinase activity of mTOR, and hence stigmasterol could serve as potential drugs for mTOR inhibition.

#### 3.5.3. ERβ

**Figure 7:**
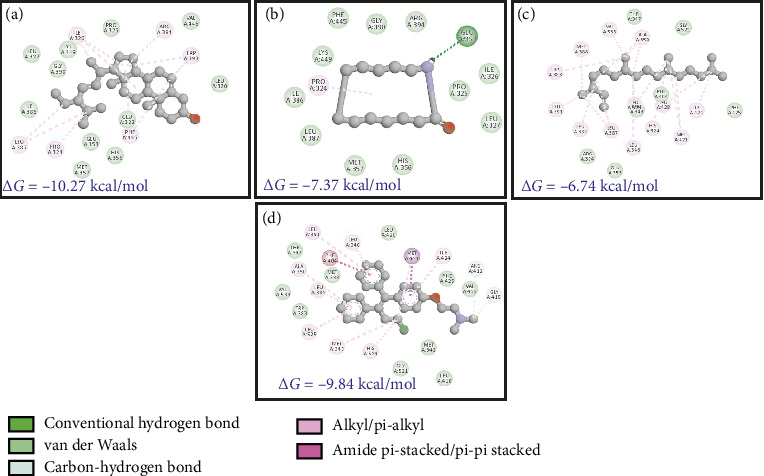
Interaction of ERβ receptor with phytochemicals. (a) SSL. (b) ACT. (c) NPD. (d) TMN.

Estrogen receptors *α* and *β* are responsible for binding to estrogen and triggering the expression of estrogen-responsive genes and both these proteins have 97% homology [70, 71]. Since ER is a nuclear receptor, it has a ligand binding domain which binds to estradiol (EDL) and then transactivates into the nucleus. Ligand-bound ER recognizes estrogen-response elements (EREs) and regulates protein kinase cascades, activation of eNOS through phosphorylation, and phosphorylation of other target proteins. Inside the nucleus, ER regulates transcription of genes responsible for endocrine, cardiovascular, and metabolic pathways, bone growth (and maturation), and skin health, apart from regulating the expression/development of secondary sexual characteristics [71]. In cardiomyocytes, ER signaling regulates vascular function and also the inflammatory response. Figures 7(a), 7(b), 7(c), and 7(d) show the binding of *B. longiflora* compounds to ERβ (3OS8) and the control compounds EDL as well as TMN. The phytochemicals were able to interact with target at two different pockets.

#### 3.5.4. Human EpidermalGrowth Factor Receptor-2 (HER-2)

HER-2, an epidermal growth factor receptor, has tyrosine kinase activity. It exhibits autophosphorylation of tyrosine residues within the cytoplasmic domain of the receptors upon dimerization of the receptor which governs the signaling pathways responsible for cell proliferation and tumorigenesis. HER-2 mutation is mainly associated with 15%–30% of breast cancers followed by 10%–30% of gastric/gastroesophageal cancers and cancer associated with the bladder, colon, endometrium, ovary, and lung [72]. In lungs, HER-2 is linked with non-small-cell lung cancer (NSCLC). Three mechanisms have been suggested for NSCLC including 1%–4% gene mutation, 2%–5% gene amplification, and 2%–30% protein overexpression [73]. Docking studies showed that stigmasterol has an effective binding affinity toward HER-2. The binding energy of stigmasterol for HER-2 is almost close to lapatinib (−7.99 and −7.72 kcal.mol^−1^, respectively). The inhibitory constant also emphasizes on higher suitability of phytochemicals as a lower dose is required.

**Figure 8:**
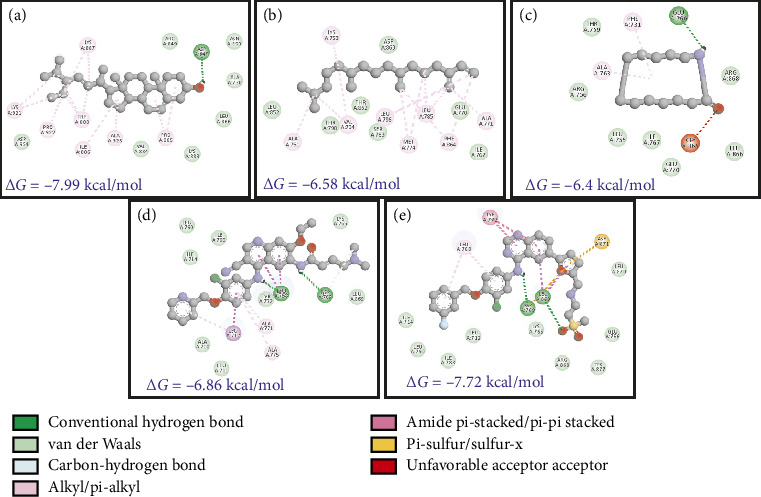
Interaction of HER-2 receptor with phytochemicals. (a) SSL. (b) NPD. (c) ACT. (d) NTB. (e) LTB.

The interaction studies and participation of different residues were also analyzed (Figures 8(a), 8(b), 8(c), 8(d), and 8(e) and Table 5). It is clear from the analysis that van der Waals forces are crucial for the interaction between target receptors and molecules (drug and phytochemical). The analysis revealed stigmasterol as one of the potent candidates for drug formulation.

## 4. Discussion

Plants are the safest and richest sources for various biomolecules and bioactive compounds [74], which played a crucial role in food and healthcare [15] for ages in different ancient and tribal practices [75]. Besides common biomolecules like carbohydrates, proteins, and lipids, plants are also rich in phytochemicals like alkaloids, tannins, and polyphenols which are well reported for healthcare and promoting abilities [76].

*Barleria longiflora* is one such species that has not been explored fully, and only a few studies are available related to its significance. The presence of diverse phytochemicals in *Barleria albostellata's* leaf and stem has been reported which contribute to its anti-inflammatory, analgesic, antihyperglycemic, antileukemic, antitumor, and antimicrobial activities [75]. Jothimuniyandi and Jayachitra [77] also reported the highest anti-inflammatory potential with ethanolic extract of *B. longiflora* leaf (56%) followed by methanol (48%) and chloroform (46%). The antioxidant activity of ethanolic extract was also maximum (83%) among all the solvents used including aqueous, chloroform, petroleum ether, methanol, ethanol, and ethyl acetate. Comparative evaluation of methanolic and aqueous extract of leaves for phytochemical analysis revealed that phytochemical diversity was higher in methanolic extract. Leaf extract contains alkaloids, saponins, tannins, flavonoids, and proteins [78].

Irregular inflammation and oxidative stress are also associated with other complications like cancer, and plant extract has shown promising results against both. Amarasiri et al. [79] reported that extracts with organic solvents have a high fraction of polyphenols and flavonoids in comparison to water and hence have higher antioxidant activity. *Barleria* extract also helped in countering the DOX-induced oxidative stress by modulating the activity of glutathione peroxidase and glutathione reductase enzymes. Among all solvents explored, hexane-based extract has improved glutathione peroxidase activity by 111% while water-based extract only has 55%. It has been suggested that treatment with the *Barleria prionitis* extracts suppressed TNF-α and IL-1β significantly. In the rat model, the anti-inflammatory potential and TNF-α, and IL-1β suppression was in the order of aqueous > butanol > ethyl acetate > hexane [79]. Panchal et al. [80] also evaluated the anticancer potential of *Barleria prionitis* against different cell lines including breast, lung, colon, and respective metastatic cells. The maximum inhibition against cancer cells reported was 76.97% against colon metastatic cells followed by 71% against breast cancer cells. In comparison, the anticancer potential was higher in current work (65%). Alkahtani et al. [81] reported viability of L929, MCF-7, and A549 cells by MTT assay against the methanolic extract of *Barleria hochstetteri*. The IC_50_ value for methanolic extract was 144.30 μg/mL (A549) and 219.67 μg/mL (MCF-7). For L929 cells, IC_50_ was much higher which suggested that methanol extract was less toxic against the noncancerous L929 cell line. It was suggested that extract activated caspase-3 to initiate apoptosis by inhibiting Bcl-2 protein.

In the current work, a total of 38 compounds have been detected including stigmasterol, resorcinol, and phenol derivatives as major population. Alkahtani et al. [81] also identified palmitic and linolenic acid derivatives and solanesol as major compounds in the methanolic extract of *Barleria hochstetteri*. GC-MS identified 9 major compounds from the extract. Gangaram et al. [75] also confirmed that decanoic acid and its derivate were one of the most prominent groups of phytochemicals in *B. albostellata* leaves and stem extract in different solvents. Alcoholic extract (methanol) has confirmed the presence of stigmasterol in methanolic extract of stem as well as leaves. It was also observed that phytochemical diversity increased in alcoholic extract in comparison to chloroform and hexane or other nonpolar as well as aqueous extract. Therefore, the activity was also much higher in similar extracts.

## 5. Conclusions

Plants are the biggest and most eco-friendly source of bioactive compounds with the least chances for side effects. *Barleria* is one of such genera carrying a significant role in traditional activities, food, and healthcare. Different species of *Barleria* have been explored for bioactivities and diverse phytochemicals; however, *B. longiflora* has only a few reports in records. In the present work, *B. longiflora* leaves from the Siriya Kalvarayan hills region were evaluated for antioxidant, anti-inflammatory, and anticancer (lung cancer). Ethanolic leaf extract has shown considerable scavenging activity against DPPHo and ABTSo+ that is compatible with previous literature. Docking studies suggested that some of the phytochemicals could be more effective in inhibiting lung cancer–associated receptors and may prove effective in finding safer and more effective drugs for cure.

## Figures and Tables

**Table 1:** Preliminary phytochemical screening of *B. longiflora.*

Name of the test	Aqueous	Methanol	Ethanol	Acetone	Chloroform	Hexane
Triterpenoids	−	+	+	−	−	−
Sugar	+	+	+	+	+	+
Catechin	−	−	−	−	+	−
Flavonoids	−	−	+	+	+	−
Saponin	+	+	−	−	+	+
Tannins	+	+	−	−	−	−
Anthraquinone	−	−	+	−	+	−
Amino acid	−	−	−	−	−	−
Sterol	−	−	+	−	−	−

**Table 2:** Qualitative analysis of *B. longiflora* leaf extract.

Test	Quantity (mg/g)
Chlorophyll a	10.12 ± 0.4
Chlorophyll b	11.22 ± 1.1
Total chlorophyll	16.25 ± 0.1
Total carotenoids	4.22 ± 2.0
Total sugar	151 ± 0.3
Total protein	1.7 ± 2.1
Total lipids	121 ± 1.5
Total free amino acids	0.7 ± 0.4
Total phenol	79.51 ± 1.1
Total tannin	55.78 ± 0.2
Total flavonoids	162 ± 0.8

**Table 3:** Chemical profiling of *B. longiflora* ethanolic leaf extract by GC-MS.

Peak	RT	Area (%)	Compound name	Molecular weight (g/mol)	Molecular formula	Medicinal properties	Reference
1	5.31	0.39	1,2-Cyclopentanedione	98.10	C_5_H_6_O_2_	No significant report found	—
2	10.208	0.5	4H-Pyran-4-one	144.12	C_6_H_8_O_4_	Antioxidant activity	[39]
3	11.537	0.43	Resorcinol	110.11	C_6_H_6_O_2_	Cytotoxic and anti-inflammatory activity	[40]
4	11.978	0.75	2,3-Dihydro-benzofuran	110.11	C_6_H_6_O_2_	Antibacterial, antidermatophytic action, and antioxidant activity	[41]
5	12.178	0.26	3,4-Anhydro-d-galactosan	144.12	C_6_H_8_O_4_	No significant report found	—
6	13.067	0.51	2H-Pyran-2-one,	130.14	C_6_H_10_O_3_	No significant report found	—
7	14.108	0.41	2-Methoxy-4-vinylphenol	150.17	C_9_H_10_O_2_	Anticancer activity, anti-inflammatory	[42]
8	16.916	0.69	Indole-6-carboxaldehyde	145.16	C_9_H_7_NO	Anticancer activity	[43]
9	17.089	0.32	Phenol	219.20	C_9_H_9_N_5_O_2_	No significant report found	—
10	18.509	0.34	Trichloroacetic acid, tridec-2-ynyl ester	314.7	C_15_H_23_Cl_3_O_2_	No significant report found	—
11	19.201	0.23	Azacyclotridecan-2-one	197.32	C_12_H_23_NO	No significant report found	—
12	19.386	0.54	3-tert-Butyl-4-hydroxyanisole	180.24	C_11_H_16_O_2_	No significant report found	—
13	19.455	0.45	Benzoic acid	168.15	C_8_H_8_O_4_	No significant report found	—
14	19.918	0.28	Diethyl phthalate	222.24	C_2_H_14_O_4_	Antioxidant	[44, 45]
15	20.779	2.0	1,3,4,5-Tetrahydroxycyclohexanecarboxylic acid	192.17	C_7_H_2_O_6_	No significant report found	—
16	22.753	0.47	Coniferyl alcohol	180.20	C_10_H_12_O_3_	Inhibits fungal growth	[46]
17	23.147	0.68	Myristic acid	228.37	C_14_H_28_O_2_	No significant report found	—
18	24.514	0.85	Neophytadiene	278.5	C_20_H_38_	Anti-inflammatory activity	[47]
19	24.63	0.17	3,7,11,15-Tetramethyl-2-hexadecene	280.5	C_20_H_40_	No significant report found	—
20	25.247	0.27	3,7,11,15-Tetramethyl-2-hexadecen-1-OL	296.5	C_20_H_40_O	No significant report found	—
21	25.999	0.24	Methyl palmitate	270.5	C_17_H_34_O_2_	Anti-inflammatory activity and anticancer effect	[48]
22	26.263	2.52	Palmitoleic acid	254.41	C_16_H_13_O_2_	Anti-inflammatory activity	[49]
23	26.646	11.69	Palmitic acid	256.42	C_16_H_32_O_2_	Anti-inflammatory activity, cytotoxic and apoptosis potential on breast cancer cells	[50]
24	27.546	0.52	Oleic acid	282.5	C_18_H_34_O_2_	Antioxidant, anti-inflammatory activity, antimicrobial activity, and anticancer effect	[51]
25	28.672	0.34	Methyl linoleate	294.5	C_18_H_34_O_2_	No significant report found	—
26	28.767	0.5	Linolenic acid, 2-hydroxy-1-(hydroxymethyl)ethyl ester	352.5	C_21_H_36_O_4_	No significant report found	—
27	29.365	16.84	Linoleic acid	280.4	C_18_H_32_O_2_	Anti-inflammatory activity	[52]
28	34.562	1.65	Hexadecanoic acid, 2-hydroxy-1-(hydroxymethyl)ethyl ester	330.5	C_19_H_38_O_4_	No significant report found	—
29	34.675	0.88	Dihydroselarene	—	—	No significant report found	—
30	34.836	8.09	Phthalic acid	166.13	C_8_H_6_O_4_	Antimicrobial activity and antioxidant	[53]
31	35.066	0.39	Cholesterol	386.7	C_27_H_46_O	Antimicrobial activity, anticancer, and antioxidant effect	[54–56]
32	36.792	0.59	Linoleic acid propyl ester	322.5	C_21_H_38_O_2_	No significant report found	—
33	36.89	1.34	cis,cis,*cis*-7,10,13-Hexadecatrienal	234.38	C_16_H_26_O	No significant report found	—
34	37.161	0.65	Glyceryl monostearate	358.6	C_21_H_42_O_4_	Antimicrobial and anticancer activity	[57, 58]
35	37.255	0.33	Alpha-Kessyl acetate	280.4	C_17_H_28_O_3_	No significant report found	—
36	38.291	4.13	Solanesol	631.1	C_45_H_74_O	Anti-inflammatory activity	[59]
37	38.968	0.27	Stigmasterol	412.7	C_29_H_48_O	Anticancer activity, anti-inflammatory activity, and antimicrobial activity	[60, 61]
38	39.625	0.18	2,2,4-Trimethyl-3-(3,8,12,16-tetramethyl-heptadeca-3,7,11,15-tetraenyl)-cyclohexanol	428.7	C_30_H_52_O	No significant report found	—

**Table 4:** B. *longiflora* phytochemical interaction with residues.

Protein targets	Compounds	Van der Waals/conventional hydrogen bond	Alkyl/pi-alkyl	Pi-sigma/pi-donor hydrogen bond/unfavorable donor-donor	Carbon hydrogen bond/covalent bond/unfavorable acceptor acceptor	Pi-pi T-shaped/pi-anion/pi-lone pair
A. 5NGB (PI3K)	Azacyclotridecan-2-one	Asp589, Met387, Arg389, Cys590, Gly593, Leu583, Phe587, Val618, Asp584, Phe585, Tyr621, Pro588, and Ser623	—	—	Glu622	—
	Stigmasterol	Pro173, Cys815, Leu735, Gln795, Gln792, Gly814, Asp736, Asp642, Gln170, and 606	Phe_609_, Leu_816_, Phe_646_, His_650_, and Met_788_	—	—	—
	Neophytadiene	Arg389, Glu622, Ser623, Gly593, Asp626, Asp584, and Glu628	Leu583, Pro588, Val618, Phe585, Tyr621, Met387, Cys590, Cys627, and Tyr	—	—	—
	Taselisib	Arg_246_, Glu_248_, Cys_815_, Lys_642_, Phe_646_, Gln_795_, Gln_610_, Gln_792_, Tyr_261_, Gln_260_, and Tyr_813_	Phe_609_, Leu_791_, and Met_788_	—	Leu_735_, Gln_170_, Ser_738_, Gly_814_, and Ser_264_	Asp_736_
	Dactolisib	Ser_754_, Asp_897_, Pro_758_, Lys_779_, Asp_787_, Thr_833_, Tyr_813_, Val_828_, and Phe_908_	Met_752_, Ile_825_, Ile_777_, and Val_827_	Ile_910_, Met_900_, and Trp_760_	Asp_911_, Asp_832_, and Ser_831_	—

B. 4JT6 chain A (mTOR)	Stigmasterol	Asp_2212_, Leu_2208_, Asn_2211_, Glu_2409_, Glu_2405_, and Asn_1898_	Pro_2213_, His_2410_, and Val_2406_	—	—	—
	Azacyclotridecan-2-one	Cys2243, Leu2354, Leu2185, Ile2237, Tyr2225, Gly2238, Val2240, and Met2345	Ile2356 and Trp2239	—	—	—
	2-Methoxy-4-vinylphenol	Arg2348, Gly2351, Ser2307, Trp2304, Leu2303, and Arg2316	Lys2306, Trp2313, Arg2317, Leu2346, and Tyr2320			
	Rapamycin	Ser_2221_, Lys_2352_, Ala_2226_, Asp_2347_, Ser_2350_, Ile_2222_, Asn_2206_, Gln_2223_, Pro_2241_, Val_2240_, Tyr_2225_, Trp_2239_, Gly_2238_, Phe_2182_, Pro_2141_, Thr_2143_, and Gly_2142_	Phe_2184_	—	Arg_2224_	—
	Ridaforolimus	Arg_2251_, Arg_2348_, Trp_2239_, Thr_2164_, Gln_2261_, Ser_2342_, Asp_2252_, Ala_2248_, Asp_2244_, Thr_2249_, Leu_2354_, and Val_2162_	Val_2240_, Met_2345_, Ile_2237_, Leu_2185_, and Ile_2356_,	—	Cys_2243_ and Ile_2163_	—

C. 3OS8 chain A (ER-beta)	Stigmasterol	Met_357_, His_356_, Glu_353_, Glu_323_, Ile_386_, Leu_320_, Gly_390_, Leu_327_, Lys_449_, Pro_325_, and Val_446_	Arg_394_, Ile_326_, Trp_393_, Phe_445_, Pro_324_, and Leu_387_	—	—	—
	Azacyclotridecan-2-one	Phe445, Gly390, Arg394, Lys449, Ile326, Pro325, Ile386, Leu387, Pro325, Leu327, and Glu353	Pro324	—	—	—
	Neophytadiene	Thr347, Gly521, Phe404, Met343, Phe425, Arg394, and Glu353	Val533, Ala350, Met388, Trp383, Leu391, Leu384, Leu346, Leu428, Ile424, Met421, and His524	—	—	—
	Toremifene	Leu_428_, Phe_425_, Val_422_, Met_388_, Thr_347_, Val_533_, Trp_383_, Met_342_, Leu_410_, and Gly_521_	Leu_391_, Leu_346_, Ala_350_, Leu_384_, Ile_424_, Leu_525_, Met_343_, and His_524_	Phe404 and Met421	Arg412 and Gly415	—

D. 3PP0 chain A (HER-2)	Stigmasterol	Asp_845_, Arg_849_, Asn_850_, Ala_730_, Leu_866_, Val_884_, Lys_883_, and Asp_924_	Lys_887_, Lys_921_, Trp_888_, Pro_922_, Ala_928_, Ile_886_, and Pro_885_	—	—	—
	Neophytadiene	Asp863, Leu852, Thr862, Thr798, Ser783, Glu770, and Ile767	Lys753, Ala751, Val734, Leu796, Leu785, Met774, Phe864, and Ala771	—	—	—
	Azacyclotridecan-2-one	Phr759, Arg756, Arg868, Leu755, Ile767, Glu770, Leu866, and Glu766	Phe731 and Ala763	—	Gly865	—
	Lapatinib	Leu_869_, Asp_769_, Leu_870_, Ile_714_, Leu_790_, Leu_712_, Ile_788_, Lys_765_, Glu_766_, Arg_868_, and Tyr_877_	Leu_768_	Tyr_772_	—	—
	Neratinib	Leu_790_, Ile_788_, Ile_714_, Lys_765_, Tyr_772_, Leu_768_, Asp_769_, Leu_869_, Ala_710_, and Leu_711_	Ala_771_ and Ala_775_	Leu_712_	—	—

**Table 5:** Docking studies of *B. longiflora* phytochemicals.

Protein targets	Ligands	Binding energy (kcal/mol)	Ligand efficiency	Inhib_constant (μM)	IE	VDE	EE
A. (PI3K) 5NGB	Azacyclotridecan-2-one	−8.43	−0.6	0.665	−8.43	−8.41	−0.01
	Stigmasterol	−8.06	−0.27	1.23	−9.85	−9.74	−0.11
	Neophytadiene	−7.12	−0.36	6.09	−10.99	−10.99	0.0
	2,3-Dihydro-benzofuran	−5.87	−0.65	50.2	−5.87	−5.8	−0.06
	2-Methoxy-4-vinylphenol	−5.67	−0.52	70.22	−6.56	−6.54	−0.02
	1,2-Cyclopentanedione	−4.84	−0.69	283.22	−4.84	−4.66	−0.18
	Resorcinol	−4.45	−0.56	548.65	−5.04	−4.92	−0.12
	3,4-Anhydro-d-galactosan	−4.09	−0.41	1000	−4.39	−4.36	−0.03
	Taselisib	−9.34	−0.27	0.143	−10.83	−10.59	−0.24
	Dactolisib	−11.36	−0.32	0.004	−12.38	−12.19	−0.18

B. (mToR) 4JT6	Stigmasterol	−11.64	−0.39	0.002	−13.43	−13.34	−0.09
	Azacyclotridecan-2-one	−7.09	−0.51	6.3	−7.09	−7.03	−0.06
	2-Methoxy-4-vinylphenol	−6.05	−0.55	36.63	−6.95	−6.82	−0.12
	2,3-Dihydro-benzofuran	−5.55	−0.62	85.93	−5.55	−5.46	−0.09
	Neophytadiene	−5.35	−0.27	120.45	−9.22	−9.23	0.0
	Resorcinol	−4.61	−0.58	418.0	−5.21	−4.78	−0.42
	1,2-Cyclopentanedione	−4.54	−0.65	468.58	−4.54	−4.46	−0.08
	3,4-Anhydro-d-galactosan	−4.37	−0.44	629.69	−4.67	−4.36	−0.32
	Rapamycin	−9.73	−0.15	0.074	−12.41	−11.97	−0.44
	Ridaforolimus	−9.07	−0.13	223.69	−12.06	−11.98	−0.07

C. (ERβ) 3OS8	Stigmasterol	−10.27	−0.34	0.0029	−12.06	−12.07	0.01
	Azacyclotridecan-2-one	−7.37	−0.53	3.96	−7.37	−7.29	−0.08
	Neophytadiene	−6.74	−0.34	11.54	−10.61	−10.61	0.0
	2,3-Dihydro-benzofuran	−5.5	−0.61	93.0	−5.5	−5.22	−0.28
	2-Methoxy-4-vinylphenol	−5.4	−0.49	111.02	−6.29	−5.89	−0.4
	Resorcinol	−4.48	−0.56	524.29	−5.07	−4.78	−0.29
	1,2-Cyclopentanedione	−4.47	−0.64	532.41	−4.47	−4.0	−0.46
	3,4-Anhydro-d-galactosan	−4.25	−0.43	767.84	−4.55	−4.34	−0.2
	Toremifene	−9.84	−0.34	0.061	−12.53	−12.42	−0.11

D. (HER-2) 3PP0	Stigmasterol	−7.99	−0.27	1.39	−9.78	−9.62	−0.16
	Neophytadiene	−6.58	−0.33	15.13	−10.45	−10.46	0.0
	Azacyclotridecan-2-one	−6.4	−0.46	20.39	−6.4	−6.26	−0.14
	2,3-Dihydro-benzofuran	−5.06	−0.56	196.8	−5.06	−5.01	−0.04
	1,2-Cyclopentanedione	−5.05	−0.72	198.64	−5.05	−4.9	−0.15
	2-Methoxy-4-vinylphenol	−5.2	−0.47	153.22	−6.1	−6.07	−0.03
	Resorcinol	−4.37	−0.55	627.91	−4.97	−4.69	−0.29
	3,4-Anhydro-d-galactosan	−4.02	−0.4	1130	−4.32	−3.92	−0.4
	Neratinib	−6.86	−0.17	9.4	−10.14	−9.18	−0.96
	Lapatinib	−7.72	−0.19	2.2	−11.0	−10.16	−0.84

## Data Availability

The data are disclosed in the manuscript. More information can be provided by the corresponding authors upon request.
